# Attenuation of Congenital Portosystemic Shunt Reduces Inflammation in Dogs

**DOI:** 10.1371/journal.pone.0117557

**Published:** 2015-02-06

**Authors:** Michael S. Tivers, Ian Handel, Adam G. Gow, Victoria J. Lipscomb, Rajiv Jalan, Richard J. Mellanby

**Affiliations:** 1 Department of Veterinary Clinical Sciences, Royal Veterinary College, Hatfield, Hertfordshire, United Kingdom; 2 Royal (Dick) School of Veterinary Studies and The Roslin Institute, The University of Edinburgh, Roslin, Midlothian, United Kingdom; 3 UCL Institute for Liver and Digestive Health, UCL Medical School, London, United Kingdom; 4 MRC Centre for Inflammation Research, University of Edinburgh, Edinburgh, United Kingdom; Oregon State University, UNITED STATES

## Abstract

Liver disease is a major cause of morbidity and mortality. One of the most significant complications in patients with liver disease is the development of neurological disturbances, termed hepatic encephalopathy. The pathogenesis of hepatic encephalopathy is incompletely understood, which has resulted in the development of a wide range of experimental models. Congenital portosystemic shunt is one of the most common congenital disorders diagnosed in client owned dogs. Our recent studies have demonstrated that the pathophysiology of canine hepatic encephalopathy is very similar to human hepatic encephalopathy, which provides strong support for the use of dogs with a congenital portosystemic shunt as a naturally occurring model of human hepatic encephalopathy. Specifically, we have demonstrated an important role for ammonia and inflammation in the development of hepatic encephalopathy in dogs with a congenital portosystemic shunt. Despite the apparent importance of inflammation in driving hepatic encephalopathy in dogs, it is unclear whether inflammation resolves following the successful treatment of liver disease. We hypothesized that haematological and biochemical evidence of inflammation, as gauged by neutrophil, lymphocyte and monocyte concentrations together with C-reactive protein concentrations, would decrease following successful treatment of congenital portosystemic shunts in dogs. One hundred and forty dogs with a congenital portosystemic shunt were enrolled into the study. We found that the proportion of dogs with a monocyte concentration above the reference range was significantly greater in dogs with hepatic encephalopathy at time of initial diagnosis. Importantly, neutrophil and monocyte concentrations significantly decreased following surgical congenital portosystemic shunt attenuation. We also found a significant decrease in C-reactive protein concentrations following surgical attenuation of congenital portosystemic shunts. Our study demonstrates that haematological and biochemical indices of inflammation reduce following successful treatment of the underlying liver disorder.

## Introduction

Hepatic encephalopathy (HE) is a neuropsychiatric syndrome, affecting people suffering from significant liver disorders [1]. The nature and severity of HE varies including impaired psychomotor function, memory and concentration and in severe cases confusion, stupor, coma and death [2]. The precise pathogenesis of HE is poorly understood although ammonia is well recognized to play an important role [2,3]. A variety of other factors have been implicated in the pathogenesis of HE, particularly inflammation, with systemic inflammatory response syndrome (SIRS) and sepsis being a common trigger for overt HE [2,3]. Circulating inflammatory cytokines including TNFα, IL-1β and IL-6 are implicated in a variety of roles in potentiating HE [2]. Studies have shown direct correlations between IL-6 and TNFα and the severity of HE and hyperammonemia [4–7]. Inflammation, in the absence of concurrent infection, has been associated with more severe HE in people with liver disease [8].

Since the biology of HE remains incompletely understood, a wide range of murine, canine and porcine experimental models has been described to facilitate further studies into the pathophysiology of HE. Our recent studies have demonstrated that client owned dogs with a congenital portosystemic shunt (cPSS) are an underutilized and potentially very informative model for human HE. cPSS are extremely rare in people [9]. However, the condition is more common in dogs, with affected animals making up 0.5% of admissions to North American veterinary teaching hospitals in 2001 and with a reported prevalence of 0.1–2.9% in pedigree dog breeds in the United Kingdom [10,11]. A cPSS allows blood from the splanchnic viscera to bypass the liver resulting in portal vein hypoperfusion and hence liver hypoplasia and hepatic insufficiency. Therefore affected animals frequently show signs of HE, including lethargy, disorientation, stupor and seizures.

Similar to earlier findings in humans, our previous work has shown associations between inflammation and HE in dogs with cPSS. We demonstrated that both increased ammonia and SIRS score were predictive of the presence of HE in dogs with cPSS [12]. In another study c-reactive protein (CRP) concentrations were significantly increased in dogs with HE associated with cPSS [13]. Furthermore increased plasma IL-6 concentrations have been associated with cPSS [14]. These data suggest that inflammation plays an important role in the development of HE in dogs, which mirrors the situation in humans. In a study of dogs treated surgically for cPSS the mean white blood cell count was increased above the reference range and this was associated with post-operative outcome [15]. However, this study did not relate the haematological findings to HE and changes in white blood cell count following surgery were not reported.

The recommended treatment for dogs with cPSS is surgical attenuation of the abnormal vessel, to redirect portal blood flow to the liver [16]. It is well recognized that successful surgical attenuation of a cPSS results in resolution of clinical signs and improvement in liver function, portal blood flow and liver volume [17–20]. We have also shown associations between markers of hepatocyte replication and liver development in dogs with cPSS, supporting a role for liver regeneration in the improvements seen post-operatively [21].

One of the great advantages of using dogs with a spontaneous cPSS as a model of HE is that we can investigate how metabolic profiles change following treatment of the underlying liver disease and subsequent resolution of HE. As dogs with a spontaneous cPSS are treated by a surgical procedure, metabolic changes can be examined without the background of complex medical and surgical treatments, which regularly confound human studies.

In dogs with a cPSS, we predicted that the diversion of the portal blood flow away from the liver and into the systemic circulation results in a systemic inflammatory response that plays an important role in the development of HE. We also predicted that attenuation of the cPSS and improved vascularization of the liver would result in reduction in inflammatory profile. Consequently, the aim of this study was to compare haematological and biochemical markers of inflammation, specifically neutrophil, lymphocyte and monocyte concentrations together with CRP concentrations, before and after surgical cPSS attenuation. We found that the proportion of dogs with a monocyte concentration above the reference range was significantly increased in dogs with HE at initial assessment. Crucially, neutrophil and monocyte concentrations and CRP concentrations significantly decreased following successful surgical treatment of the cPSS. Our study demonstrates that haematological and biochemical evidence of inflammation reduces following successful treatment of a cPSS in dogs.

## Methods

Consecutive cases of dogs with a cPSS diagnosed at the Royal Veterinary College (RVC), London which had a haematology profile performed at initial presentation were considered eligible for inclusion in the study. All animals were treated routinely according to their clinical presentation and the data was gathered retrospectively from the clinical records. The diagnostic criteria for a cPSS were the identification of a portosystemic shunt on intra-operative mesenteric portovenography and by visual identification of the shunting vessel during an exploratory celiotomy. The presence or absence of HE at the time of blood sampling and clinical examination was ascertained by reviewing the case notes. The dog was considered to have HE if it had clinical signs of lethargy, inappropriate behaviour, disorientation, circling, head pressing or seizures. If the dog had no clinical signs of neurological impairment or dysfunction it was considered to not have HE. Each dog underwent suture attenuation of the cPSS as previously described [18,22]. Dogs that did not tolerate complete attenuation, due to intra-operative portal hypertension, had partial suture attenuation. Dogs treated with partial attenuation had repeat surgery approximately three months post-operatively and attenuation was completed where possible.

Blood samples were taken from cPSS dogs pre-operatively and at follow up examination for diagnostic purposes and, where available, residual blood was prospectively collected for the study. Details of each dog’s haematology profile were recorded at the initial examination and at re-examination, following surgical attenuation. Haematology was performed using an Abbott Cell-Dyn 3500 (Abbott Diagnostics Limited, Berkshire, UK) as previously described [23]. At least a 100-white blood cell manual differential count was undertaken to establish the concentrations of neutrophils, monocytes, lymphocytes, eosinophils and basophils. Blood smears were evaluated under the direct supervision of a Board-certified veterinary clinical pathologist in every case. The serum was separated and stored at -80°C. Serum CRP before and after cPSS attenuation was measured using an immunoturbidimetric assay as previously described [13].

Continuous data were reported as medians with 25%-75% interquartile range in brackets. The lymphocyte, monocyte, eosinophil, neutrophil and basophil concentrations, as well as age, were compared in dogs with and without HE at initial examination and between dogs with extrahepatic and intrahepatic cPSS by a Mann Whitney U test. The haematology parameters and CRP concentrations were compared before and after surgery by a Wilcoxon paired test. Categorical variables were reported as proportions and comparisons were undertaken using a Fisher’s exact test. Categorical variables at initial examination and at follow up were compared with a mid-*p* McNemar test. For all tests significance was set at the 5% level (p = 0.05).

The Royal Veterinary College Ethics and Welfare Committee granted ethical approval and owners gave full, informed consent.

## Results

### Signalment

One hundred and forty dogs were eligible for inclusion in the study. This was an expanded cohort from that previously reported [12]. Forty-two were entire females (30.0%), 21 neutered females (15.0%), 54 entire males (38.6%) and 23 neutered males (16.4%). Their ages ranged from 57 days to nearly 12 years with a median age of 318 days. The breeds of the dogs included in the study are shown in Table 1. Twenty-nine dogs (20.7%) had an intrahepatic cPSS and 111 dogs (79.3%) had an extrahepatic cPSS. Fifty-three dogs (37.9%) had complete attenuation and 87 (62.1%) had partial attenuation during surgical treatment of cPSS. Follow-up blood tests were performed on 85 dogs that were subsequently free of HE. Two dogs had persistent HE at follow-up and were excluded from the results. Of the 85 dogs 59 (69.4%) were receiving medical management of HE (combination of restricted protein diet, antibiotics, lactulose) whereas 26 (30.6%) were not on any treatment for HE. The median time between blood sample at initial examination and post-attenuation sample was 109 days (96–138). Follow-up haematology was available for 21/29 (72.4%) intrahepatic dogs and 64/111 (57.7%) extrahepatic dogs. This difference was not statistically significant (p = 0.07). Follow-up haematology was available for 27/53 (50.9%) dogs that had a complete attenuation and for 58/87 dogs (66.7%) that had a partial attenuation at first surgery. This difference was not statistically significant (p = 0.20).

**Table 1:** Table showing the number of dogs for each pedigree breed and crossbreeds included in the study.

Breed of Dog	Number included in study
Basset Hound	2
Bichon Frise	5
Border Collie	2
Border Terrier	3
Cairn Terrier	4
Cavalier King Charles Spaniel	1
Chihuahua	2
Cocker Spaniel	5
Crossbreed	11
Flat Coat Retriever	1
Golden Retriever	4
Great Dane	1
Hovawart	1
Irish Setter	3
Irish Water Spaniel	1
Jack Russell Terrier	9
Labradoodle	1
Labrador Retriever	9
Lhasa Apso	2
Maltese Terrier	1
Miniature Dachshund	1
Miniature Poodle	2
Miniature Schnauzer	10
Norfolk Terrier	5
Papillon	1
Pug	4
Rhodesian Ridgeback	1
Scottish Terrier	1
Shetland Sheepdog	2
Shih Tzu	10
Springer Spaniel	1
Staffordshire Bull Terrier	1
Tibetan Terrier	1
Weimaraner	1
West Highland White Terrier	18
Yorkshire Terrier	13

Thirty-seven dogs (26.4%) had HE and 103 (73.6%) had no HE at initial diagnosis. For the dogs with HE 10 were entire females (27.0%), eight neutered females (21.6%), 14 entire males (37.8%) and five neutered males (13.5%). For the dogs without HE 32 were entire females (31.1%), 13 neutered females (12.6%), 40 entire males (38.8%) and 18 neutered males (17.5%). There was no significant difference between sex (p = 0.70) and neuter status (p = 0.68) between the two groups. For the dogs with HE the median age was 262 days (117–749), which was not significantly different (p = 0.26) from the median age for dogs without HE at 320 days (163–799). For the dogs with HE 29 (78.4%) had an extrahepatic cPSS and eight (21.6%) had an intrahepatic cPSS. For the dogs without HE 82 (79.6%) had an extrahepatic cPSS and 21 (20.4%) had an intrahepatic cPSS. There was no significant difference in the type of shunt between the two groups (p = 1.0). For the dogs with HE five (13.5%) had complete attenuation and 32 (86.5%) had partial attenuation during surgical treatment of cPSS. This was statistically significantly different from the dogs without HE, of which 48 (46.6%) had complete attenuation and 55 (53.4%) had partial attenuation (p<0.001). For the dogs with HE 14 (37.8%) were receiving medical management of HE (combination of restricted protein diet, antibiotics, lactulose) and 23 (62.2%) were not receiving any treatment. For dogs without HE 75 (72.8%) were receiving medical management and 28 (27.2%) were not receiving any treatment. This difference was statistically significant (p<0.001).

### Haematology

The neutrophil, monocyte, lymphocyte, eosinophil and basophil concentrations in dogs with and without HE at initial diagnosis are reported in Table 2. Twenty-two of 103 (21.4%) dogs without HE had a neutrophil concentration above the reference range compared to 14 out of 37 (37.8%) dogs with HE (p = 0.07). A significantly greater proportion of dogs with HE had a monocyte concentration above the reference range compared to dogs without HE (12/103–11.7% dogs without HE, 12/37–32.4% dogs with HE, p = 0.009). White blood cell concentrations in dogs with intrahepatic cPSS and extrahepatic cPSS at initial diagnosis are reported in Table 3.

**Table 2:** Median and interquartile range of white blood cell parameters in dogs with congenital portosystemic shunts (cPSS) with and without hepatic encephalopathy (HE) at initial diagnosis.

	No HE (n = 103)	HE (n = 37)	P value
Neutrophil	9.15 (6.59–11.00)	10.19 (7.23–15.27)	0.11
Monocyte	0.82 (0.57–1.25)	0.91 (0.50–1.84)	0.35
Lymphocyte	2.90 (1.87–4.22)	2.44 (1.78–3.36)	0.11
Eosinophil	0.58 (0.18–1.02)	0.55 (0.24–1.07)	0.90
Basophil	0.00 (0.00–0.00)	0.00 (0.00–0.00)	0.49

**Table 3:** Median and interquartile range of white blood cell parameters in dogs with extrahepatic and intrahepatic congenital portosystemic shunts (cPSS).

	Extrahepatic (n = 111)	Intrahepatic (n = 29)	P value
Neutrophil	9.15 (7.14–11.75)	9.29 (6.66–11.40)	0.98
Monocyte	0.82 (0.52–1.26)	0.91 (0.62–1.39)	0.64
Lymphocyte	2.7 (1.83–4.21)	2.68 (1.90–3.66)	0.67
Eosinophil	0.51 (0.17–0.89)	0.82 (0.41–1.69)	0.005
Basophil	0.00 (0.00–0.00)	0.00 (0.00–0.00)	0.45

Follow up haematology was available on 59/103 dogs (57.3%) that did not have HE at initial diagnosis and remained free of HE at post-treatment evaluation. The neutrophil concentration significantly decreased post-treatment (pre median of 9.45 (7.31–11.53), post median of 7.19 (5.75–9.18), p<0.001). The monocyte concentration also decreased from a median of 0.86 (0.61–1.26) to a median of 0.59 (0.40–0.79) (p = 0.005). There was no significant change in lymphocytes (p = 0.28), eosinophils (p = 0.09) and basophils (p = 0.47). In the dogs that did not have HE at initial diagnosis there was a significant decrease in the proportion of dogs that had a neutrophil concentration above the reference range after treatment (neutrophil 15/59 pre, 6/59 post, p = 0.031). There was no significant difference in the proportion of dogs that had a monocyte concentration above the reference range following treatment (monocyte 7/59 pre, 5/59 post, p = 0.508).

Follow up haematology was available on 26/37 dogs (70.3%) that had HE at initial diagnosis and were subsequently free of HE at post-treatment evaluation. There was no significant difference between the proportion of dogs with HE at initial diagnosis that had follow up compared with the proportion of dogs without HE that had follow-up (p = 0.18). The neutrophil concentration significantly decreased post-treatment (pre median of 9.73 (7.11–15.68), post median of 7.35 (5.21–8.54), p = 0.002). The monocyte concentration also decreased from a median of 0.74 (0.48–1.99) to a median of 0.61 (0.42–0.90) (p = 0.022). There was no significant change in lymphocytes (p = 0.53), eosinophils (p = 0.34) and basophils (p = 0.63). In the dogs that had HE at initial diagnosis there was a significant decrease in the proportion of dogs that had neutrophil or monocyte concentration above the reference range after treatment (neutrophil 10/26 pre, 2/26 post, p = 0.004; monocyte 8/26 pre, 1/26 post, p = 0.021).

There was no significant difference in post-treatment neutrophil (p = 0.77) or monocyte (p = 0.63) concentrations in dogs, which had initially presented with HE compared to dogs that were free of HE at initial presentation.

### C-reactive protein

C-reactive protein was measured in a sub-group of 19 dogs before and at follow-up examination after cPSS attenuation. The median time interval between samples was 110 days (range 97–139). Six of these dogs (31.6%) had HE at initial examination and 13 (68.4%) did not have HE. No dogs had clinical signs of HE at follow-up. Eighteen dogs (94.7%) had extrahepatic cPSS and one (5.3%) had an intrahepatic cPSS. Thirteen dogs (68.4%) tolerated complete cPSS attenuation at surgery and six dogs (31.6%) only tolerated partial attenuation.

The median CRP concentration significantly decreased following cPSS attenuation (pre median of 3.40 (0.73–12.73) mg/l, post median of 1.43 (0.59–3.14) mg/l, p = 0.03).

## Discussion

The central finding of this study was that inflammation was significantly associated with the presence of HE in dogs with cPSS and that inflammation significantly reduced following successful surgical treatment. Consistent with our previous studies which demonstrated a role for inflammation in the development of canine HE [12–14,24], we demonstrated that proportionally more dogs with HE have a monocyte concentration above the reference range than those without HE. Unsurprisingly, dogs that did not show signs of HE on examination were significantly more likely to be receiving medical treatment. Importantly, the longitudinal nature of this study allowed us to track haematological and biochemical markers of inflammation following treatment of the underlying liver disorder. We discovered that neutrophil and monocyte counts decreased following treatment in dogs with a cPSS. These findings were further supported by a significant reduction in the concentration of CRP following successful treatment. Collectively, these observations demonstrate that successful treatment of liver disease in dogs results in a decrease in biochemical and haematological markers of inflammation.

These findings are similar to numerous studies in human HE which have also demonstrated the importance of inflammation in the development of HE in patients with liver disorders. Whilst ammonia is well accepted as playing a key role in the pathogenesis of HE, there is a growing evidence base that inflammation is also important [3]. Pro-inflammatory cytokines such as TNFα, IL-1β and IL6 are increased in people with HE [2] and inflammation has been shown to increase the susceptibility of the brain to the effects of hyperammonaemia [2]. Several studies have shown a correlation between serum IL-6 concentration and the severity of HE in people with liver disease [4,5,7,8]. We have previously shown that IL-6 is significantly increased in dogs with cPSS compared to control dogs [14]. The source of the increased concentration of IL-6 in dogs with cPSS is unclear. Our current finding that the concentrations of monocytes, which are capable of producing large amounts of IL-6, significantly decrease following surgery in both HE and no HE groups suggests that these cells may be important in producing pro-inflammatory cytokines in dogs with a cPSS.

Generalized inflammation may be associated with liver disease for several reasons. Lipopolysaccharide (LPS) is absorbed from the gut and delivered to the liver for clearance by Kupffer cells via the portal vein [25–27]. Lipopolysaccharide concentrations in the peripheral and portal circulation are increased in people with liver cirrhosis due to increased absorption from the gut and decreased hepatic clearance [28–31]. Hepatic clearance is reduced either due to the shunting of blood past the liver via multiple acquired shunts (MAS) or impaired LPS clearance due to underlying liver pathology [28]. Plasma LPS concentrations have been shown to be increased in dogs with both cPSS and experimentally created MAS, similar to the situation in people [32, 33]. We have also shown that the hepatic expression of toll-like receptor 4 (TLR4), which binds LPS, is significantly associated with the degree of liver development, the degree of portal blood flow and the response to surgery in dogs with cPSS, supporting a role for LPS in these dogs [33]. Impaired hepatic function is associated with increased serum IL-6 concentrations in people with liver disease [4,5,34]. Similarly we have shown that plasma IL-6 concentrations are increased in dogs with cPSS [14].

The finding that neutrophil and monocyte concentrations decrease following resolution of HE indicates a potential role for the innate immune system in the development of HE in dogs. A study of the role of inflammation in people with liver cirrhosis demonstrated increased neutrophil concentrations in people with minimal hepatic encephalopathy (MHE) [8]. Minimal hepatic encephalopathy is a condition where individuals have no overt clinical signs of HE but do demonstrate significant abnormalities on psychometric testing and in neurophysiological performance [8,35]. In addition, the current study showed that that neutrophil and monocyte counts significantly decreased after successful cPSS attenuation. These data demonstrate that hematological and biochemical evidence of inflammation is reduced following surgery, in concert with clinical improvement and resolution of HE. These findings support the concept that inflammation plays an important role in HE in dogs. cPSS attenuation improves portal blood flow and is associated with improved hepatic function. Intuitively, we can suggest that improved hepatic blood flow results in increased clearance of LPS and therefore reduced inflammation [33]. The resolution of inflammation in the current study supports this concept.

Interestingly, whilst neutrophil and monocyte counts significantly decreased following surgery overall, there was a significant decrease for both cPSS dogs with HE and those without. This would suggest that there is underlying inflammation present in the majority of dogs with cPSS whether they are exhibiting obvious signs of HE or not. Therefore some dogs with cPSS may be suffering from occult HE and this could result in their under treatment. This situation could mirror MHE seen in some humans with liver cirrhosis [8,35]. It is unknown whether MHE is a phenomenon in dogs and more detailed cognitive testing is required to address this issue. It is important to recognize that we did not have follow-up haematology samples from all of the dogs included in the study. However, there was no significant difference in the proportion of dogs with haematology follow-up based on the type of cPSS (intrahepatic / extrahepatic) and degree of attenuation (partial / complete). This suggests that the sub-group of dogs with haematology follow-up is broadly representative of the entire cohort.

The group of dogs with HE did not significantly differ from the group without HE in terms of age, sex, neuter status and type of cPSS. However, dogs without HE were significantly more likely to be able to tolerate a complete attenuation at first surgery compared to those with HE. It has been shown that dogs that can tolerate a complete attenuation have a significantly greater portal blood flow [18]. Intuitively we can suggest that these dogs may have less shunting of portal blood and hence may have reduced clinical signs compared with dogs with reduced portal blood flow. This would fit with the findings of the current study.

We also found that CRP significantly decreased following cPSS attenuation. It would have been ideal to measure CRP in all of the dogs in this study but unfortunately samples were only available for the 19 dogs that were included in this part of the study. CRP is frequently used as a marker of inflammation in dogs, although it is relatively non-specific [36]. We have previously shown that CRP is significantly increased in cPSS dogs with HE compared to those without HE and normal dogs [13]. It is also recognized that CRP is increased in people with liver cirrhosis and MHE [8]. The fact that CRP decreases following successful surgery provides further support for the important role of inflammation in dogs with cPSS. The measurement of additional markers of inflammation before and after cPSS attenuation, such as IL-6 and TNFα, would have provided additional information. Unfortunately, this was not possible from this cohort of dogs.

In summary, our findings add to our previous work by demonstrating that haematological and biochemical evidence of inflammation decrease follow surgical correction of a cPSS. Our findings further demonstrate the value of dogs with a cPSS as a spontaneous model of human HE.
